# Identification of microRNAs associated with glioma diagnosis and prognosis

**DOI:** 10.18632/oncotarget.14445

**Published:** 2017-01-02

**Authors:** Xinyun Ye, Wenjin Wei, Zhengyu Zhang, Chunming He, Ruijin Yang, Jinshi Zhang, Zhiwu Wu, Qianliang Huang, Qiuhua Jiang

**Affiliations:** ^1^ Department of Neurosurgery, The Affiliated Ganzhou Hospital of Nanchang University, Ganzhou, Jiangxi 341000, China

**Keywords:** glioma, miRNA, diagnosis, prognosis, meta-analysis

## Abstract

The sensitivity and specificity of microRNAs (miRNAs) for diagnosing glioma are controversial. We therefore performed a meta-analysis to systematically identify glioma-associated miRNAs. We initially screened five miRNA microarray datasets to evaluate the differential expression of miRNAs between glioma and normal tissues. We next compared the expression of the miRNAs in different organs and tissues to assess the sensitivity and specificity of the differentially expressed miRNAs in the diagnosis of glioma. Finally, pathway analysis was performed using GeneGO. We identified 27 candidate miRNAs associated with glioma initiation, progression, and patient prognosis. Sensitivity and specificity analysis indicated miR-15a, miR-16, miR-21, miR-23a, and miR-9 were up-regulated, while miR-124 was down-regulated in glioma. Ten signaling pathways showed the strongest association with glioma development and progression: the p53 pathway feedback loops 2, Interleukin signaling pathway, Toll receptor signaling pathway, Parkinson's disease, Notch signaling pathway, Cadherin signaling pathway, Apoptosis signaling pathway, VEGF signaling pathway, Alzheimer disease-amyloid secretase pathway, and the FGF signaling pathway. Our results indicate that the integration of miRNA, gene, and protein expression data can yield valuable biomarkers for glioma diagnosis and treatment. Indeed, six of the miRNAs identified in this study may be useful diagnostic and prognostic biomarkers in glioma.

## INTRODUCTION

MicroRNAs (miRNAs) are single stranded, endogenous, non-coding RNAs of approximately 22 nucleotides. The first miRNA was discovered in 1993 and named *lin-4*. This gene was found to control the timing of *C. elegans* larval development [1]. However, miRNAs were not recognized as a distinct class of biological regulators with conserved functions until the second miRNA, *let-7*, was identified in 2000 [2, 3]. Since then, thousands of miRNAs have been identified in *Homo sapiens*. These molecules have diverse biological functions in glioma initiation and progression [4–8]. More than half of all miRNAs inhibit target gene expression by binding to complementary sequences in the 3’ untranslated regions of mRNAs [9, 10]. A single miRNA may have hundreds of mRNA targets, and a single gene may have hundreds of miRNA regulators. Approximately 30% of all protein-coding genes may be regulated by miRNAs.

Glioma is an extremely aggressive and lethal type of brain tumor that arises from glial cells [11–16]. Therefore, early diagnosis and treatment is critical. Dysregulation of miRNAs has been shown to promote tumorigenesis through inhibition of tumor suppressor genes or inappropriate activation of oncogenes [17–26]. For example, miRNA-21 (miR-21) enhances the chemotherapeutic effects of taxol on human glioblastoma multiforme cells [27]. Zhang et al. identified nine miRNAs associated with survival in 82 glioblastoma patients [28]. Additionally, Piwecka et al. identified many new miRNAs that were differentially expressed in malignant glioma tissue, and Drusco et al. identified miRNAs that were differentially expressed in cerebrospinal fluid, which could be used to diagnose central nervous system malignancies [29, 30]. Along with recent advances in microRNA technology, the number of miRNAs with diagnostic and prognostic value has increased. These biomarkers could improve the rapid and accurate diagnosis of glioma [31]. Several studies have achieved conflicting results, which could be explained by differences in the miRNA profiling systems and analytic platforms used in the studies.

Although the expression of individual miRNAs may be useful for distinguishing between cancer types, the potential of miRNAs as biomarkers for glioma requires a systematic analysis of the existing data. Therefore, we performed a meta-analysis to determine whether specific miRNAs could differentiate between glioma and normal tissue, and whether these miRNAs could be used as diagnostic or prognostic biomarkers. Additionally, we investigated the genes and pathways targeted by these miRNAs.

## RESULTS

### MiRNA expression profiling

We downloaded glioma miRNA datasets from the Gene Expression Omnibus (GEO), a public database at the National Center for Biotechnology Information. We manually screened the studies to identify those that included miRNA arrays and false discovery rate (FDR) and fold-change (FC) calculations. Five studies met the inclusion criteria and were included in our meta-analysis (Table 1). All studies were published between 2012 and 2015. Three of the studies were performed in China, one was performed in the United States, and one was performed in Poland. A total of 147 glioma and 34 normal tissue samples were included in the meta-analysis. There were 68,362 miRNAs that were reported to be differentially expressed in glioma compared to normal tissue. The study details are shown in Table 1.

**Table 1:** Glioma miRNA expression profiling data

Author and Accession Number	Institution	Total samples	Sample information		MicroRNA Number	Year
			Normal	Glioma		
Zhang WGSE25631	Capital Medical University, China	87	5	82	1146	2012
Chen WGSE44726	Nanjing Medical University, China	12	6	6	62976	2013
Piwecka MGSE61710	Warsaw University of Life Sciences, Poland	17	5	12	909	2015
Drusco AGSE62381	The Ohio State University, USA	58	14	44	753	2015
Yang JGSE65626	Capital Medical University, China	6	3	3	2578	2015

### Predictive value of miRNA expression in glioma

**Figure 1:**
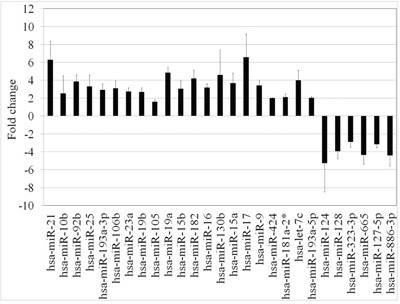
Meta-analysis of differentially expressed miRNAs A total of 27 miRNAs were differentially expressed between glioma and normal tissue. Of these miRNAs, 21 were up-regulated and six were down-regulated.

Glioma histological subtypes are diagnosed pathologically. Because the datasets were collected using different platforms, the probe sequences were mapped to miRBase (http://www.mirbase.org) using BLAST tools to identify concordant miRNA names. To determine whether the expression of the miRNAs could be used to distinguish between glioma and control cases, we performed a meta-analysis of three primary datasets (GSE25631, GSE61710, and GSE62381). These datasets comprised the training cohort and contained 118 cancer and 24 control tissue samples. The validation cohort consisted of two additional miRNA datasets (GSE65626 and GSE44726). We analyzed miRNA expression profiles in these five glioma microarray datasets compared to normal controls. The miRNAs that were differentially expressed in various tissues are shown in Table 2. Microarray datasets were normalized using a normalization algorithm in GeneSpring 13.0 (Agilent). Normalization removed batch effects. We identified 27 miRNAs that were differentially expressed between normal and malignant tissue (Figure 1). Of these miRNAs, miR-124, miR-128, miR-323-3p, miR-665, miR-127-5p, and miR-886-3p were down-regulated, while miR-21, miR-10b, miR-92b, miR-25, miR-193a-3p, miR-106b, miR-23a, miR-19b, miR-105, miR-19a, miR-15b, miR-182, miR-16, miR-130b, miR-15a, miR-17, miR-9, miR-424, miR-181a-2, let-7c, and 193a-5p were up-regulated in glioma tissue.

**Table 2:** Differential expression of miRNAs in various tissues

Hsa-miRNA	Liver	Ovary	Uterus	Prostate	Brain	Glioma
let-7c	0.006	0.022	0.036	0.045	0.003	0.009
miR-10b	<0.001	0.003	0.005	<0.001	<0.001	0.002
miR-105	<0.001	0.002	<0.001	<0.001	<0.001	0.002
miR-106b	<0.001	<0.001	<0.001	<0.001	<0.001	<0.001
miR-124	<0.001	<0.001	<0.001	<0.001	0.227	<0.001
miR-127-5p	<0.001	<0.001	<0.001	<0.001	<0.001	<0.001
miR-128	<0.001	<0.001	<0.001	<0.001	<0.001	<0.001
miR-130a	0.002	0.001	<0.001	<0.001	0.001	<0.001
miR-15a	0.002	0.005	0.003	0.004	0.019	0.044
miR-15b	0.004	0.002	0.002	0.002	<0.001	0.001
miR-16	0.034	0.044	0.036	0.056	0.048	0.127
miR-17	0.001	<0.001	0.002	<0.001	<0.001	<0.001
miR-181a	0.005	0.007	<0.001	0.002	0.027	0.027
miR-182	0.002	<0.001	0.002	<0.001	<0.001	0.002
miR-19a	0.003	<0.001	0.002	<0.001	<0.001	0.003
mir-19b	<0.001	0.002	<0.001	0.001	0.001	0.002
miR-193a-3p	0.001	<0.001	<0.001	0.002	<0.001	0.002
miR-193a-5p	0.004	0.002	<0.001	<0.001	<0.001	<0.001
miR-21	0.001	0.004	0.023	0.022	0.005	0.156
miR-23a	0.001	0.005	0.007	0.008	0.001	0.037
miR-25	0.001	0.001	0.003	0.002	<0.001	0.003
miR-323-3p	<0.001	<0.001	<0.001	<0.001	<0.001	<0.001
miR-424	<0.001	<0.001	<0.001	<0.001	<0.001	<0.001
miR-665	0.001	<0.001	<0.001	<0.001	0.001	<0.001
miR-886-3p	<0.001	0.002	<0.001	<0.001	0.001	<0.001
miR-9	0.004	0.002	0.002	0.001	0.204	0.263
miR-92b	0.004	0.003	0.002	0.002	0.001	0.002

### Sensitivity and specificity of differentially expressed miRNAs

We obtained expression data for differentially expressed miRNAs in the liver, ovary, uterus, brain, and glioma tissue from the GEO database. We found that miR-15a, miR-16, miR-21, miR-23a, and miR-9 were up-regulated while miR-124 was down-regulated in glioma compared to normal tissue (Table 2).

**Figure 2:**
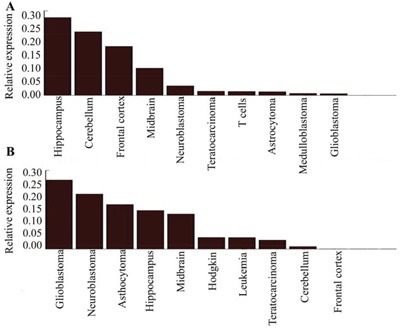
Relative expression of miR-124 **(A)** and miR-9 **(B)** compared to GAPDH in various tissues. The highest miR-124 expression is observed in the hippocampus followed by the cerebellum, cerebral cortex, and midbrain. The expression is lower in various types of glioma. Higher miR-9 expression is observed in glioblastoma and neuroblastoma tissue compared to normal and astrocytoma tissue.

Interestingly, miR-124 and miR-9 were selectively expressed in neural tissues. The highest miR-124 expression was observed in the hippocampus followed by the cerebellum, cerebral cortex, and midbrain. Decreased miR-124 expression was observed in various types of glioma including neuroblastoma, astrocytoma, medulloblastoma, and glioblastoma (Figure 2A). Finally, the highest miR-9 expression was observed in glioblastoma and neuroblastoma, followed by astrocytoma and normal brain tissue (hippocampus and midbrain) (Figure 2B).

**Figure 3:**
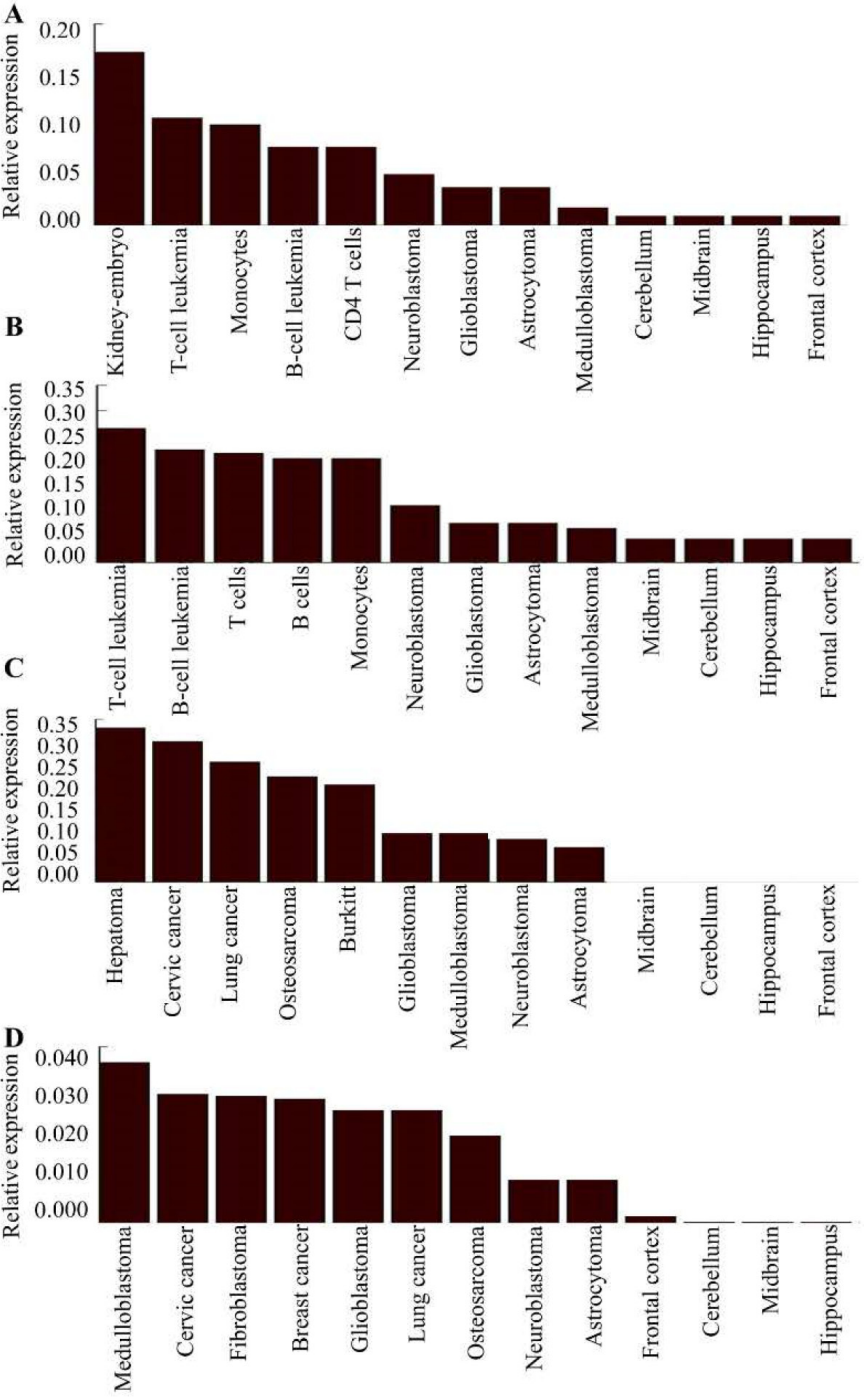
Relative expression of miR-15a **(A)** miR-16 **(B)** miR-21 **(C)** and miR-23a **(D)** compared to GAPDH in various tissues. MiR-15a and miR-16 are predominantly expressed in lymphocytes and monocytes. MiR-21 is highly expressed in various types of cancer cells including hepatocellular carcinoma, HeLa, lung, and osteosarcoma cells. The expression of miR-23a is increased in various types of cancers including glioma, HeLa, and breast cancer cells.

MiR-15a, miR-16, miR-21, and miR-23a are non-specific miRNAs that are expressed in many tissues. The expression of miR-15a and miR-16, which are primarily expressed in lymphocytes and monocytes, was higher in glioma compared to normal brain tissue (Figure 3A and 3B). MiR-21 was highly expressed in all cancer cells evaluated including hepatocellular carcinoma, ovarian cancer, lung cancer, and osteosarcoma. In contrast, low levels were observed in normal brain tissue (Figure 3C). The expression of miR-23a was higher in various cancer cells (e.g. glioma, HeLa, and breast cancer cells) compared to normal brain tissue (Figure 3D).

### MiRNA target prediction and functional analysis

**Figure 4:**
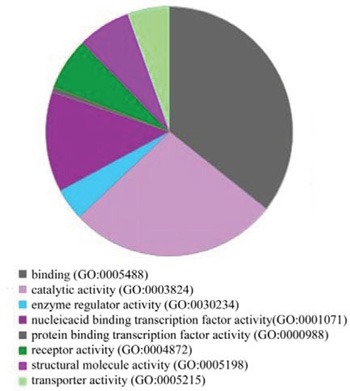
GO analysis of target gene functions

MiRNAs regulate various biological processes through inhibition of target gene expression. To identify potential miRNA target genes, we queried the three most popular computational databases, miRBase, microRNA, and TargetScan, to identify target genes reported in all three databases. We identified 1,204 genes predicted to be targeted by six miRNAs. We next performed gene ontology (GO) analysis in order to define the biological functions of the target genes in a broad range of biological processes (e.g. catalytic activity, enzyme regulatory activity, nucleic acid-binding transcription factor activity, protein-binding transcription factor activity, receptor activity, structural molecule activity, and transporter activity) (Figure 4).

We performed KEGG pathway analysis of the target genes in various tissues. The top 10 pathways identified were the following: the p53 pathway feedback loops 2, Interleukin signaling pathway, Toll receptor signaling pathway, Parkinson disease, Notch signaling pathway, Cadherin signaling pathway, Apoptosis signaling pathway, VEGF signaling pathway, Alzheimer disease-amyloid secretase pathway, and the FGF signaling pathway. These 10 pathways were highly associated with glioma initiation and progression (Table 3).

**Table 3:** The top 10 enriched pathways based on GeneGO analysis

Pathways	Components	-log (p-value)
p53 pathway feedback loops 2	32	10.1
Interleukin signaling pathway	36	7.16
Toll receptor signaling pathway	46	5.33
Parkinson disease	37	5.31
Notch signaling pathway	23	5.30
Cadherin signaling pathway	16	5.24
Apoptosis signaling pathway	72	5.16
VEGF signaling pathway	25	5.03
Alzheimer disease-amyloid secretase pathway	31	4.55
FGF signaling pathway	26	4.37

## DISCUSSION

In our study, we used publicly available miRNA datasets to evaluate whether specific miRNAs may be useful and accurate biomarkers for discriminating between glioma and normal tissue. We selected five microarray datasets and systematically identified promising miRNAs that could distinguish between glioma and control tissue. We found that 27 microRNAs were differentially expressed between glioma and normal tissue. Six of these microRNAs had more accurate predictive value in distinguishing glioma from control tissue (i.e. higher sensitivity, higher specificity, and statistical significance.

The expression patterns and functions of various miRNAs in glioma are diverse. Some miRNAs such as miR-124 and miR-9 are specifically expressed in the brain. MiR-124, which is highly expressed in the central nervous system including the hippocampus, cerebellum, cerebral cortex, and midbrain, stimulates neuronal differentiation to maintain embryonic stem cell self-renewal and pluripotency [32]. In high-grade malignant gliomas and astrocytomas, miR-124 was either minimally expressed or absent. Loss of miR-124 enhances the stem-like traits and invasiveness of glioma cells [32, 33]. Cai et al. found that down-regulation of miR-124 resulted in an increase in phosphorylated FAK, MMP2, vimentin, and N-cadherin levels in U87 cells through CAPN4, and that miR-124 suppressed the migration and invasion of glioma cells *in vitro* via CAPN4 [34]. Lu et al. reported that miR-124 inhibited glioma cell proliferation and invasion by blocking IQGAP1 expression and downstream activation of β-catenin and cyclin D1 [35]. Shi et al. demonstrated that down-regulation of miR-124 in tumor tissue promoted glioma development, angiogenesis, and chemoresistance, suggesting that miR-124 may be a useful diagnostic marker and therapeutic target in glioma [36]. Interestingly, miR-124 inhibited the migration and invasion of glioma cells through down-regulation of ROCK1, SOS1, CDK4, STAT3, and PPP1R13L expression [37–40], indicating miR-124 may be a valuable biomarker for glioma. The brain-enriched miR-9 also has been implicated in nervous system development and other physiological and pathological processes in several organisms. Increased expression of miRNA-9 was associated with an unfavorable prognosis in human glioma [41]. However, other studies have presented conflicting results. For example, suppression of miRNA-9 by mutant EGFR signaling resulted in up-regulation of FOXP1 and enhanced glioblastoma tumorigenicity [42–43].

Some of the miRNAs are not brain-specific. We found that miR-15a, miR-16, miR-21, and miR-23 were highly expressed in various cancer tissues including glioma. MiRNAs can also function as tumor suppressors. For example, Xie et al. demonstrated that down-regulation of miR-15a was associated with an adverse prognosis in human glioma patients [44]. Yang et al. validated the role of miR-16 as a tumor suppressor in glioma and uncovered a novel mechanism of miR-16-mediated inhibition of glioma growth and invasiveness through inhibition of BCL2 and the NF-κB1/MMP-9 signaling pathway [45, 46]. These results indicate that increased expression of miR-15a and miR-16 is protective against glioma. Some miRNAs may be oncogenic. For example, miR-23a promoted the invasion of U251 and U87 cells, at least in part by directly targeting HOXD10 and modulating the expression of MMP-14 [47, 48]. Moreover, the oncogenic miR-23a promotes glioma development through the cAMP response element-binding protein [49].

MiR-21 is one of the most well-studied miRNAs. It is over-expressed in various cancer tissues. Here, we found that miR-21 expression was increased in several malignant cell types (particularly hepatocellular carcinoma cells). Dysregulated miR-21 expression was observed in all types of glioma. The oncogenic miR-21 inhibits the tumor suppressive activity of FBXO11 to promote tumorigenesis [50–54]. Moreover, miR-21 promotes glioblastoma initiation through down-regulating IGFBP3 [55]. Additionally, miR-21 was shown to down-regulate the expression of the tumor suppressor PDCD4 in the human glioblastoma cell line T98G [56]. Plasma miR-21 concentration may be a useful biomarker in glioblastoma patients [57]. The oncogenic miRNAs miR-21 and miR-23a are potential therapeutic targets in glioma.

To explore the interactions between miRNAs and their corresponding target genes, we performed pathway analyses using the list of target genes referenced by all three computational databases. The top 10 significant pathways showed enrichment of 2,104 genes associated with cancer initiation and progression. These genes represented a wide range of biological processes. We took advantage of statistical tools to mine available data for each target gene. We found that the FDR and FC values for more than half of the target genes met our criteria.

Our results demonstrate that a combination of miRNA and target gene expression could enable the identification of promising biomarkers for glioma and provide novel insights into the molecular mechanisms responsible for glioma initiation and progression. Additional studies are required to validate the impact of the six miRNAs on glioma development, progression, and patient prognosis.

## MATERIALS AND METHODS

### Search strategies

A two-phase literature search was performed to identify studies involving glioma miRNA expression profiling. First, microarray datasets were extracted from the NCBI and GEO databases using the following MESH terms: (microRNA OR miRNA) AND (brain carcinoma or brain cancer or brain tumor or brain neoplasm or glioma or glioblastoma) AND (expression OR profile OR profiling). Next, references from the included studies were manually screened to identify additional relevant studies. Three reviewers independently extracted the data from all eligible studies. All sample datasets (i) were from humans, (ii) included miRNA arrays, and (iii) were part of studies that included FDR and FC calculations. The datasets analyzed in this study are summarized in Table 1.

### Data collection and processing

We collected five publicly available glioma microRNA microarray datasets that were assembled using different platforms. Each of the datasets was generated by a separate laboratory. To obtain more consistent results, we performed a meta-analysis of the multiple miRNA microarrays. The microarray datasets were analyzed based on the same statistical hypothesis (cancer versus normal tissue). We converted log2-transformed datasets from the different platforms into FC. A 5% FDR in Bayesian statistical analysis was then used to identify statistically significant differences in miRNA expression between cancer and control cases.

### Sensitivity and specificity of differentially expressed miRNAs

We analyzed publicly available expression data for miRNAs in various human tissues including the liver, ovary, uterus, prostate, and brain. We first collected data for miRNAs that were differentially expressed in these tissues, and then compared the expression to that observed in glioma tissue. The data were obtained from miRBase (www.mirbase.org), microRNA (www.microrna.org/microrna/home.do), and RNAhybrid (bibiserv2.cebitec.uni-bielefeld.de/rnahybrid). The SPSS statistical software was used analyze the diagnostic value of miRNAs that were differentially expressed between glioma and normal tissue.

### MiRNA target prediction and functional analysis

To identify potential miRNA target genes, we first queried the three most popular computational databases, miRBase [58], microRNA [59], and TargetScan [60]. We identified target genes that were present in all 3 databases. GO analysis of the potential target genes was based on the terms of the Gene Ontology database: gene function-related biological processes were detected, and genes with similar functions were combined. KEGG pathway analysis was performed to identify the pathways that were significantly associated with the target gene candidates based on a comparison with the entire set of reference genes. Target genes with a FDR ≤ 0.05 were considered to be significantly enriched.
